# Effects of Neuropeptides and Mechanical Loading on Bone Cell Resorption *in Vitro*

**DOI:** 10.3390/ijms15045874

**Published:** 2014-04-08

**Authors:** Yeong-Min Yoo, Ji Hyun Kwag, Kyung Hwan Kim, Chi Hyun Kim

**Affiliations:** Department of Biomedical Engineering, College of Health Science, Yonsei University, Wonju, Gangwon-do 220-710, Korea; E-Mails: yyeongm@hanmail.net (Y.-M.Y.); guiltyspace@yonsei.ac.kr (J.H.K.); khkim0604@yonsei.ac.kr (K.H.K.)

**Keywords:** neuropeptides, mechanical loading, bone resorption, RANKL (receptor activator of nuclear factor kappa B (NF-κB) ligand), OPG (osteoprotegerin)

## Abstract

Neuropeptides such as vasoactive intestinal peptide (VIP) and calcitonin gene-related peptide (CGRP) are present in nerve fibers of bone tissues and have been suggested to potentially regulate bone remodeling. Oscillatory fluid flow (OFF)-induced shear stress is a potent signal in mechanotransduction that is capable of regulating both anabolic and catabolic bone remodeling. However, the interaction between neuropeptides and mechanical induction in bone remodeling is poorly understood. In this study, we attempted to quantify the effects of combined neuropeptides and mechanical stimuli on mRNA and protein expression related to bone resorption. Neuropeptides (VIP or CGRP) and/or OFF-induced shear stress were applied to MC3T3-E1 pre-osteoblastic cells and changes in receptor activator of nuclear factor kappa B (NF-κB) ligand (RANKL) and osteoprotegerin (OPG) mRNA and protein levels were quantified. Neuropeptides and OFF-induced shear stress similarly decreased RANKL and increased OPG levels compared to control. Changes were not further enhanced with combined neuropeptides and OFF-induced shear stress. These results suggest that neuropeptides CGRP and VIP have an important role in suppressing bone resorptive activities through RANKL/OPG pathway, similar to mechanical loading.

## 1. Introduction

Many cytokines, present in the extracellular matrix or synthesized by the bone cells, are involved in bone-remodeling [1]. The receptor activator of nuclear factor kappa B (NF-κB) ligand (RANKL) and osteoprotegerin (OPG) signaling pathway has culminated recently in the discovery of the genetics on bone metabolism. Osteoblasts have a significant role in the control of bone resorption through release of OPG, an inhibitor of osteoclast function, and RANKL, an osteoclast differentiation factor. RANKL and its soluble receptor play a critical role during the osteoclast differentiation and act in a paracrine way [2]. Therefore, RANKL and OPG genes are important transcription factors in the regulation of bone formation and resorption for maintaining bone mass [3,4].

The differentiation and activation of osteoclasts require two key elements, macrophage colony stimulating factor (M-CSF) and RANKL [5]. M-CSF promotes the proliferation, survival of osteoclast progenitor cells. Mature osteoclast differentiation requires RANKL binding to RANK on the surface of osteoclast progenitor cells [6]. Mature osteoclasts resorb bone by secreting tartrate-resistant acid phosphatase (TRAP) to the space between cell membrane and bone matrix. TRAP is a well-known marker of bone resorption to measure the activation of osteoclasts [7,8].

Mechanical loading is an important regulator of bone cell activity. When dynamic physical load is applied to bone, the interstitial fluid that surrounds bone cells is forced out of regions of high compressive strain and then returns when the load is removed. This results in bone cells being exposed to a dynamic oscillating fluid flow (OFF)-induced shear stress. OFF-induced shear stress is a potent regulator of both anabolic and catabolic bone cell metabolism. In a recent study using a co-culture system, dynamic OFF has been demonstrated to suppress osteoclast formation by decreasing RANKL and increasing OPG mRNA expression [9].

Neuropeptides and neurotransmitters include vasoactive intestinal peptide (VIP), calcitonin gene-related peptide (CGRP), substance P (SP), pituitary adenylate cyclase activating peptide (PACAP), neuropeptide Y (NPY), leptin, somatostatin (SOM), serotonin, glutamine, norepinephrine, and tyrosine hydroxylase (TH) [10–16]. Mice lacking the Y2 receptors for NPY display an increase in appetite and body mass, resulting in higher bone trabecular volumes [17]. Leptin knockout mice results in higher bone mass due to increased bone formation [18]. Neuropeptides and neurotransmitters have been suggested to potentially regulate bone remodeling [19,20].

VIP and CGRP are present in nerve fibers of various regions within the bone tissue (e.g., periosteum, bone marrow cavity, and vascular canal) [11,13] and their receptors are expressed in MC3T3-E1 pre-osteoblastic cells [21]. VIP is a 28 amino acid peptide supported by its presence in skeletal nerve fibers in periosteum of the bone. VIP has been shown to stimulate calcium release from neonatal mouse calvariae in organ culture [22]. CGRP receptors are detected on osteoblastic cells [11].

Recently, bone formation has been shown to be neuronally regulated in distant bones of the skeleton that were not loaded [20]. However, very few studies exist with the goal to elucidate the bone remodeling process via the interaction between neuropeptides and mechanical induction. In addition, there are even fewer studies on the resorptive aspect of bone remodeling. Therefore, our goal was to quantify the effects of combined mechanical stimuli and neuropeptides on mRNA and protein expression related to bone resorption using a pre-established OFF-induced shear stress system.

## 2. Results and Discussion

### 2.1. Osteoclast Formation and Activity

**Figure 1.**
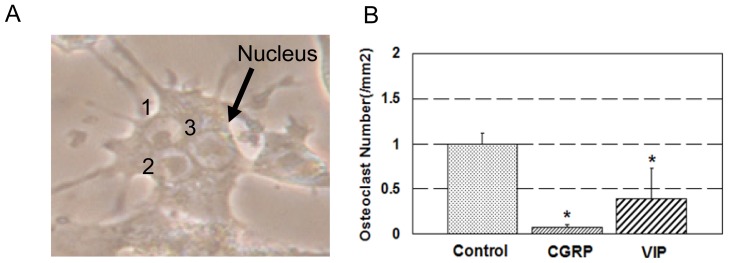
Osteoclast formation and activity. (**A**) Co-culture of MC3T3-E1 pre-osteoblastic cells and RAW 264.7 machrophage cells resulted in the formation of multinucleated cells with three or more nuclei; and (**B**) effect of CGRP (calcitonin gene-related peptide) and VIP (vasoactive intestinal peptide) on formation of osteoclasts. Using a microscope with a magnification ×200, Cells with three or more nuclei were considered to be osteoclasts. * *p* < 0.05 control. The arrow and 1, 2, 3 are nucleus.

**Figure 2.**
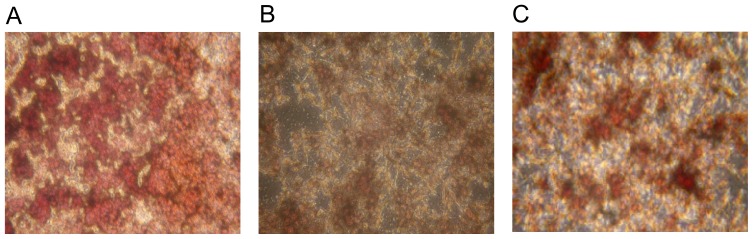
Multinucleated osteoclasts were stained red colors by TRAP (tartrate-resistant acid phosphatase) Assay Kit. (**A**) Control group (TRAP-positive multinucleated cells with no VIP or CGRP treatment); (**B**) 10 nM CGRP treatment group; and **(C**) 1 μM VIP treatment group. To assess the formation and activity of osteoclasts, cells were stained for TRAP activity on Day 9 with a magnification ×200.

Co-culture of MC3T3-E1 pre-osteoblastic cells and RAW 264.7 macrophage cells for 9 days resulted in formation of TRAP-positive multinucleated osteoclasts in the presence of M-CSF and 1α,25-dihydroxyvitamin D_3_. The number of osteoclasts was significantly decreased by 90% with the addition of CGRP and by 40% with the addition of VIP during co-culture (Figure 1). Also, TRAP activity was significantly reduced in CGRP and VIP treated groups compared to control (Figure 2).

### 2.2. Expression of RANKL and OPG mRNA

**Figure 3.**
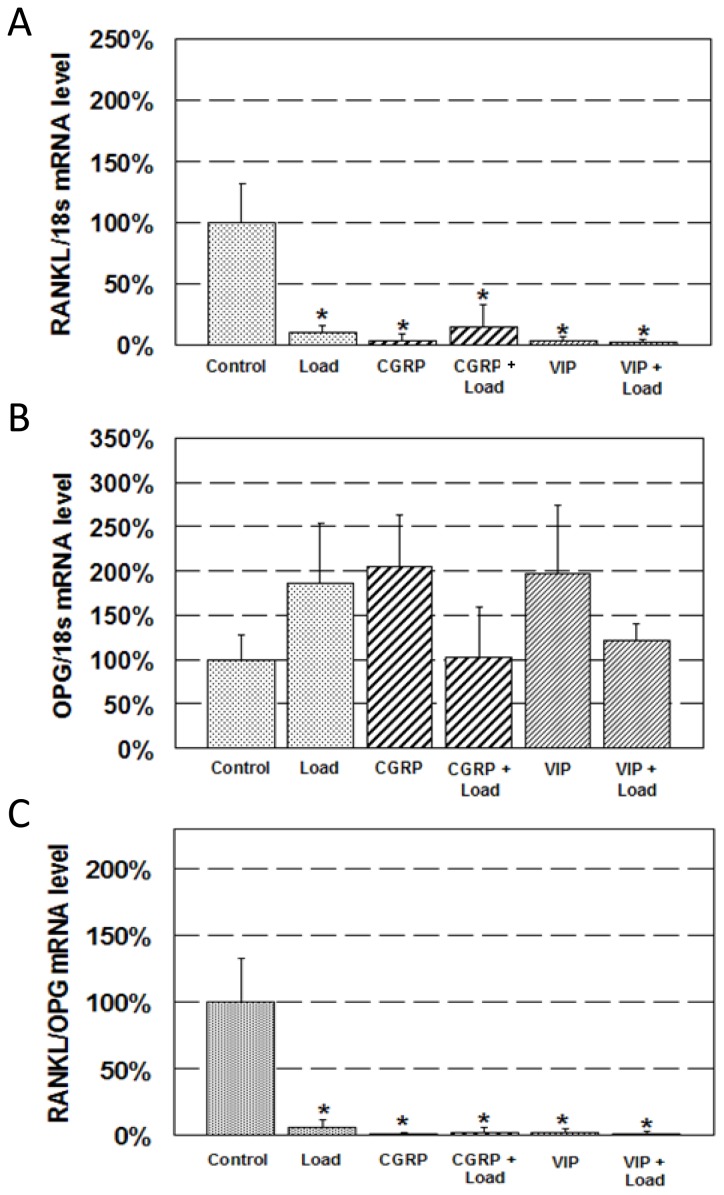
Expression of RANKL (receptor activator of nuclear factor kappa B (NF-κB) ligand) and OPG (osteoprotegerin) mRNA. Change in (**A**) RANKL mRNA; (**B**) OPG mRNA; and (**C**) RANKL/OPG mRNA ratio after neurotransmitter and/or mechanical stimulation. * *p* < 0.05 control.

Immediately after exposure to 1 h OFF-induced shear stress, RANKL mRNA decreased by 90% compared to control (Figure 3A). Treatment with neuropeptides also significantly decreased RANKL mRNA. CGRP treatment decreased RANKL mRNA by 97% and VIP treatment by 96% compared to control. Combined neuropeptide treatment and loading resulted in a similar decrease to loading or neurotransmitter only treatment groups. CGRP + Load and VIP + Load decreased RANKL mRNA by 85% and 98%, respectively, compared to control.

OPG mRNA expression did not change with loading and/or neuropeptide treatment statistically (Figure 3B). Still, there was a trend of increase in OPG mRNA with either loading or neuropeptide treatment.

RANKL/OPG mRNA ratio displayed a significant decrease in all treated groups compared to control (Figure 3C). RANKL/OPG ratio decreased in the loading group (94%), neuropeptide groups (99% in CGRP and 97% in VIP), and the combined neuropeptide and loading groups (97% in CGRP + Load and 98% in VIP + Load).

### 2.3. Expression of RANKL and OPG Protein

**Figure 4.**
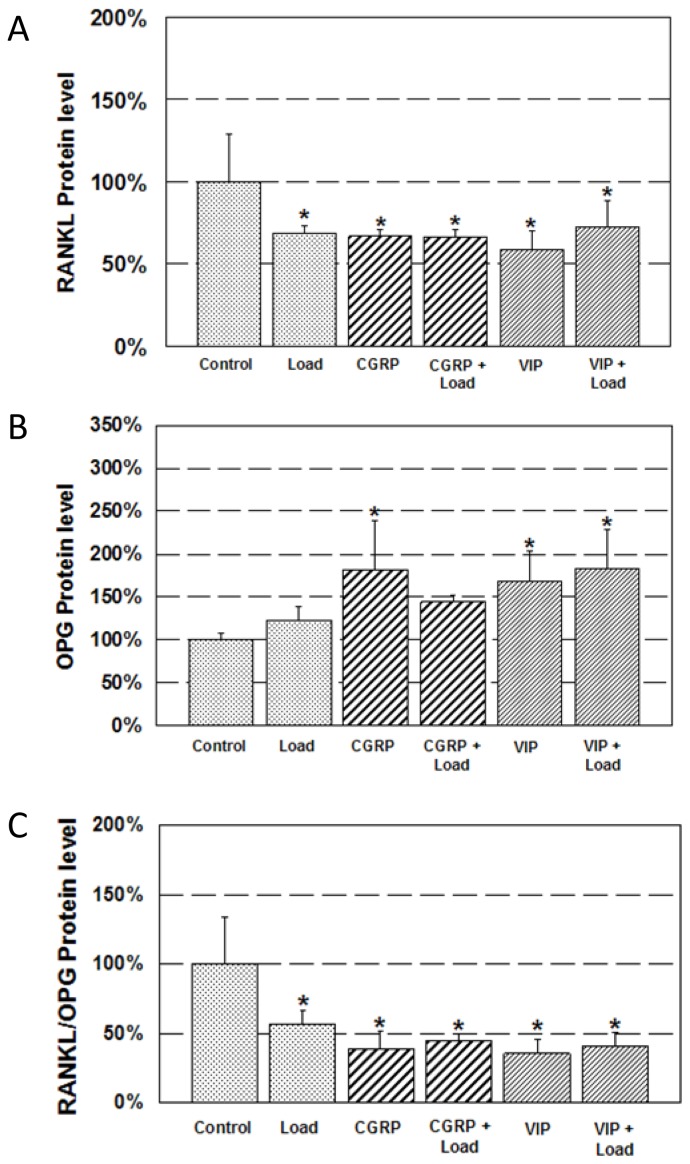
Expression of RANKL and OPG protein. Change in (**A**) RANKL protein; (**B**) OPG protein; and (**C**) RANKL/OPG protein ratio after neurotransmitter and/or mechanical stimulation. * *p* < 0.05 control.

Mechanical loading for 1 h resulted in a significant decrease (approximately 30%) in RANKL protein level (Figure 4A). Neuropeptide treatment resulted in a similar decrease. CGRP treatment and VIP treatment decreased RANKL protein level by approximately 35% and 40%, respectively. Combined CGRP and loading resulted in a 35% decrease and combined VIP and loading resulted in a 30% decrease. Similar to mRNA expression, combined neuropeptide and loading treatment did not further enhance the decrease in RANKL protein level.

Loading did not result in an increase in OPG protein level compared to control (Figure 4B). However, neuropeptide treatment significantly increased OPG protein. CGRP increased OPG protein level by 180% and VIP increased OPG protein level by 170% compared to control. Combined VIP and loading also displayed a significant increase (180%) in OPG protein level compared to control.

RANKL/OPG protein ratio significantly decreased in all treatment groups compared to control (Figure 4C). RANKL/OPG ratio resulted in a 45% decrease in the loading group, approximately 60% decreases in the CGRP and VIP treatment groups, and approximately 60% decreases in the CGRP + Load and VIP + Load groups.

### 2.4. Discussion

The question addressed by this study was whether neuropeptides have the potential to suppress bone resorptive activities in a mechanism similar to mechanical loading. The main finding from this study is that neuropeptides CGRP and VIP both suppress bone resorptive activities through regulation of the RANKL/OPG expression similar to mechanical loading.

We have shown that treatment of MC3T3-E1 pre-osteoblastic cells with neuropeptides CGRP or VIP can significantly decrease osteoclast formation and TRAP activity. These results are similar to the effects of OFF-induced shear stress on bone cells [9] and suggest that CGRP and VIP, two neuropeptides that exist in bone tissues [11,20,23], may have the potential to independently suppress bone resorption.

Suppression of bone resorptive activities with neuropeptide treatment involves the regulation of the RANKL/OPG signaling mechanism. Previous studies show decrease in bone resorptive activities with various types of mechanical loading, including OFF-induced shear stress and dynamic loads [9,24]. Results from this present study are consistent with those studies in that exposure of cells to OFF results in a significant decrease in RANKL/OPG mRNA and protein ratio. Treatment with CGRP or VIP followed a similar trend with loading in that decrease in RANKL/OPG ratio was mostly due to a decrease in RANKL mRNA and protein. A simultaneous increase in OPG resulted in a synergistic decrease in RANKL/OPG mRNA and protein ratio.

Interestingly, the extent of decrease in RANKL/OPG ratio was similar in the loading only (*i.e.*, Load), neuropeptide only (*i.e.*, CGRP and VIP), and combined neuropeptide and loading groups (*i.e*., CGRP + Load and VIP + Load). This indicates that combined neuropeptide and mechanical loading does not further enhance the decrease in RANKL/OPG ratio and subsequently the extent of suppression of bone resorption. This finding suggests that OFF-induced shear stress and neuropeptides VIP and CGRP may regulate bone resorptive activities in a similar cellular signal transduction mechanism.

The limitation of this study is that although regulation of bone resorptive activities were similar using either neuropeptides or mechanical loading, it is not possible to address whether they both result in *in vivo* bone remodeling at identical sites of bone tissue. It would be fascinating if *in vivo* suppression of bone resorption through neuropeptide treatment occurs in a manner similar to mechanical loading (*i.e.*, minimization of the decrease in bone tissue mechanical properties such as modulus and strength). Therefore, *in vivo* animal studies are necessary to understand whether neuropeptides and mechanical loading treatment both result in similar bone microstructure and strength.

In summary, we have shown that neuropeptides CGRP and VIP have an important role in suppressing bone resorptive activities through the RANKL/OPG pathway, similar to mechanical loading. Understanding the neural regulation aspect of bone remodeling and its combined effect on mechanically induced bone remodeling may have the potential to treat bone diseases.

## 3. Experimental Section

### 3.1. Osteoclast Formation and Activity

MC3T3-E1 pre-osteoblastic cells and RAW 264.7 murine monocytic macrophage cells (25000:10000 ratio) were co-cultured in 6-well tissue culture plates in alpha-MEM (GIBCO, Grand Island, NY, USA) with 10% FBS (GIBCO) and 1% penicillin/streptomycin (GIBCO). M-CSF (25 ng/mL; Peprotech, Rocky Hill, NJ, USA) and 1α,25-dihydroxyvitamin D_3_ (10 nM; Sigma-Aldrich, St. Louis, MO, USA) were added to induce the expression of RANKL and subsequently the formation of osteoclasts on Days 1, 3, 5, and 7. Neurotransmitters VIP (10 μM; Sigma-Aldrich) or CGRP (10 nM; Sigma-Aldrich) were added on Days 1, 3, 5, and 7. Cells were placed in an incubator at 37 °C and 5% CO_2_. To assess the formation and activity of osteoclasts, cells were stained for TRAP activity (Sigma-Aldrich) on Day 9. Using a microscope with a 20× objective, TRAP-positive cells with three or more nuclei were considered to be osteoclasts and counted by three blinded independent observers.

### 3.2. Oscillatory Fluid Flow (OFF)-Induced Shear Stress

MC3T3-E1 pre-osteoblastic cells were cultured on tissue culture dishes in alpha-MEM (GIBCO) with 10% FBS (GIBCO) and 1% penicillin/streptomycin (GIBCO). 1α,25-dihydroxyvitamin D_3_ (10 nM) was added to induce the expression of RANKL on Days 1 and 3. Neurotransmitters VIP (1 μM) or CGRP (10 nM) were added on Days 1 and 3. Cells were placed in an incubator at 37 °C and 5% CO_2_. On Day 5, cells were subcultured on glass slides (75 mm × 38 mm × 1 mm) at a density of approximately 7 × 10^5^ cells/cm^2^, prepared for either osteoclast formation study or OFF-induced shear stress study.

On Day 6, the slides with cells were placed in custom-built parallel plate flow chambers under sterile conditions. Dynamic OFF was produced with a glass syringe connected in series with rigid walled tubing and a parallel plate flow chamber. The syringe was driven by an actuator that can deliver a precise media flow rate at 1 Hz and a peak shear stress of ±1 Pa for 1 h. Control cells were also placed in flow chambers with no fluid flow applied. The six experimental groups were Control, Load, CGRP, CGRP + Load, VIP, and VIP + Load. Cells were then prepared for quantification of either gene expression or protein synthesis.

### 3.3. RNA Isolation and Real-time RT-PCR

After oscillatory fluid flow for 1 h, the slide with MC3T3-E1 pre-osteoblastic cells were removed from the flow chambers and placed in sterile petri dishes for RNA isolation. The cells were lysed and total RNA extracted using Tri-Reagent (Sigma-Aldrich). Real-time RT-PCR (Applied Biosystems, Foster City, CA, USA) was analyzed to show the results of RANKL and OPG gene expression (Taqman Gene Expression Assays, Applied Biosystems). The results were normalized by the housekeeping gene 18S (Taqman Gene Expression Assays, Applied Biosystems). Each RNA sample was analyzed in triplicates.

### 3.4. Protein Quantification

After oscillatory fluid flow for 1 h, the slides with MC3T3-E1 pre-osteoblastic cells were removed from the flow chambers and placed in sterile petri dishes with 10 mL fresh serum-free media and incubated at 37 °C and 5% CO_2_ for 1 h. After incubation, the supernatant samples were collected to measure RANKL and OPG protein release by ELISA using Quantikine Mouse RANKL Immunoassay and Quantikine Mouse OPG Immunoassay (R&D systems, Minneapolis, MN, USA). The MC3T3-E1 osteoblastic cells were also extracted by lysis buffer (1% Triton X-100, 0.5% Nonidet P-40, 10 mM Tris (pH 7.4), 0.2 mM PMSF, 150 mM NaCl, 1 mM EDTA, 30 mM Na_4_P_2_O_7_) to assay the total protein quantities using Quick Start™ Bradford Protein Assay (Bio-rad Laboratories, Munich, Germany).

### 3.5. Statistical Analysis

Statistical significance was determined by ANOVA followed by the post hoc Fisher’s least significant difference test. A significance level of 0.05 was employed for all statistical analyses.

## 4. Conclusions

In this study, neuropeptides VIP or CGRP and/or OFF-induced shear stress were applied to MC3T3-E1 pre-osteoblastic cells and changes in receptor activator of RANKL and OPG mRNA and protein levels were quantified. Neuropeptides and OFF-induced shear stress similarly decreased RANKL and increased OPG levels compared to control. Changes were not further enhanced with combined neuropeptides and OFF-induced shear stress. Therefore, neuropeptides CGRP and VIP have an important role in suppressing bone resorptive activities through RANKL/OPG pathway, similar to mechanical loading.
